# Puerperal uterine inversion managed by the uterine balloon tamponade

**DOI:** 10.11604/pamj.2015.22.331.7823

**Published:** 2015-12-03

**Authors:** Mariétou Thiam, Mouhamadou Mansour Niang, Lamine Gueye, Fatou Rachel Sarr, Marie Edouard Faye Dieme, Mamadou Lamine Cisse

**Affiliations:** 1 Thies University, Faculty of Health Sciences, Thiès, Senegal; 2 Service of Obstetrics and Gynecology Regional Hospital Thiès, Senegal; 3 Department of Obstetrics and Gynecology Institute of Social Hygiene in Dakar, Senegal; 4 Cheikh Anta Diop University of Dakar, Dakar, Senegal

**Keywords:** Uterine inversion, postpartum hemorrhage, uterine balloon tamponade

## Abstract

The uterine inversion is a rare and severe puerperal complication. Uncontrolled cord traction and uterine expression are the common causes described. We report a case of uterine inversion stage III caused by poor management of the third stage of labor. It was about a 20 years old primigravida referred in our unit for postpartum hemorrhage due to uterine atony. After manual reduction of the uterus, the use of intra uterine balloon tamponade helped to stop the hemorrhage. The uterine inversion is a rare complication that may cause maternel death. The diagnosis is clinical and its management must be immediate to avoid maternal complications.

## Introduction

Acute puerperal uterine inversion is the penetration into the uterine cavity of the uterus bottom. This is a rare and serious obstetrical complication because it can impact badly the maternal prognosis. We report a case of acute puerperal uterine inversion occurred after a vaginal delivery complicated by major postpartum hemorrhage and managed with the uterine balloon tamponade.

## Patient and observation

**Figure 1:**
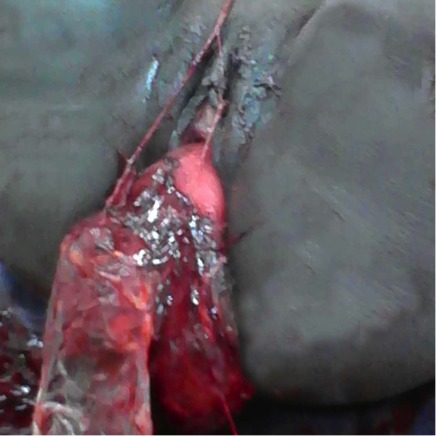
Uterine inversion stage III with the placenta adhering to the fundus

**Figure 2:**
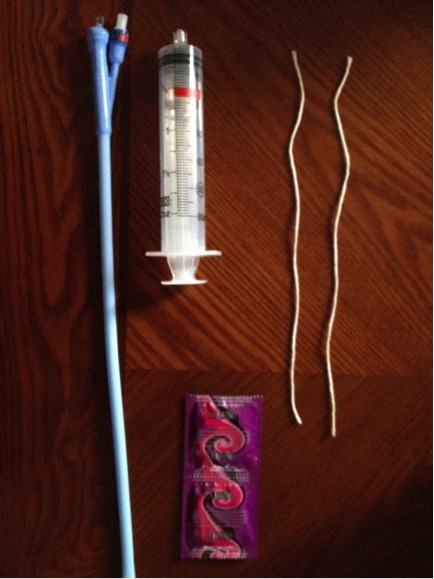
The balloon device include a catheter, a condom and a 60 ml syringe

**Figure 3:**
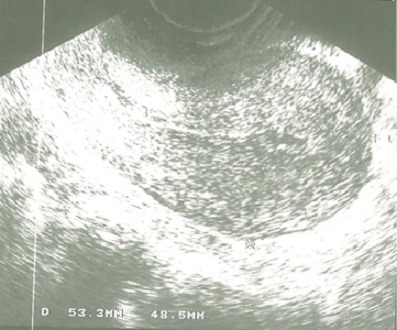
Pelvic ultrasound normal

A 20 years old primigravida, without particular medical and surgical history was admitted in our unit for cardiovascular collapse due to postpartum hemorrhage. She had a normal delivery an hour earlier in another health center. The newborn was a female weighting 2700 g with a Apgar score 10/10 in the fifth minute. The delivery was made by a matron with active management of the third stage of labor (AMTSL) using intramuscular injection of 5 IU of Oxytocin followed by uncontrolled cord traction causing the uterine inversion. One hour after the delivery, when she arrived in our unit, the examination revealed cardiovascular collapse with blood pressure at 5/2 and stunning pulse. The gynecological examination revealed a uterine inversion stage III with the placenta adhering to the fundus (Figure 1). The requested laboratory tests showed a blood Rhesus group B positive; a prothrombin rate 79,7; an hemoglobin rate 6 g / dl and a platelets rate 211000 / mm^3^. The patient was urgently transferred to the operating room. After general anesthesia, the uterus was replaced in his anatomical position simply by taxis without difficulty. The clinical examination after uterine reintegration revealed uterine atony with persistent bleeding despite the uterine massage and intravenous (IV) Oxytocin. At this moment, we took the decision to place the uterine ballon tamponade. The balloon device include a catheter, a condom and a 60 ml syringe (Figure 2). Once the condom is attached to the catheter and introduced into the uterine cavity, after we start inflating the condom with physiological saline up to 700 cc. The patient had received antibiotics (2 g of cefazolin IV), blood transfusion (1500 ml) and plasma transfusion (1000 ml). The uterine balloon tamponade was removed 8 hours later and after, the clinical examination showed that there was no bleeding and the uterus was well retracted. We administered 10 IU of Oxytocin IV, continued the antibiotic orally during 7 days. The patient stayed 5 days at the hospital without any complications. A month later, the gyneacological examination and the pelvic ultrasound was normal (Figure 3).

## Discussion

The puerperal uterine inversion is a rare and serious obstetric emergency. Its frequency is about 1/2000 deliveries in the USA and 1/20 000 deliveries in Europe [1]. Maternal death can occur in 15% of cases [2]. Some iatrogenic etiologic factors have been described: excessive traction on the umbilical cord before placental abruption, uterine expression and the sudden stop of the Oxytocin infusion [3, 4]. Primiparity, short umbilical cord and fast or long labor are also associated with this complication [1]. In our case, the risk factors was a forced traction on the cord while the placenta was not detached from the myometrium and the delivery performed by unqualified personnel. With the introduction of AMTSL, the incidence of uterine inversion may increase if the obstetrician does not respect the conditions of the directed delivery including controlled cord traction and proscription of uterine expression to prevent this complication. The diagnosis is primarily clinical with hemorrhage, pelvic pain and cardiovascular collapse which are the main warning signs. The uterine inversion stage III is visible from the perineal inspection with a fundus externalized to the vulva as a fleshy mass bloody. The treatment must be immediate and based on three main points: correction of cardiovascular collapse, intra-abdominal replacement of the uterus and uterine atony correction. Literature data recommends first a manual reduction by simply reversing taxis which consists to push the fundus in order to replace it or the Johnson's method which consists of a pressure on the vaginal cul-de-sac by vaginal fingers and the bottom by the palm of the hand. [1] In case of failure of the manual repositioning, other methods are described: - hydrostatic methods which consist in filling the vagina with saline in sealing the vagina either manually (O′Sullivan) or by using a suction cup (Ogueh) [1, 3, 5] - the uterine packing [3] - surgical correction by high way by the Huntington technique or Haultain or vaginally with the technique of Spinelli [1] - hysterectomy hemostasis remains the gold standard in case of persistent bleeding but is exceptionnel [6]. More recently the use of balloons such as Rusch or Bakri have been described in the literature as the re-inversion prevention method within two hours following initial reintegration [4, 7–10]. They are associated with fewer complications (infection and necrosis uterine) compared to uterine sutures. In our case, despite uterine massage and Oxytocin infusion, there was a persistent atony. The use of intrauterine balloon tamponade allowed us to stop the bleeding and prevent uterine re-inversion. This balloon, easy to use and low cost, has the advantage of being available in our resource-limited countries. Whatever the technique used, antibiotic therapy should be systematic to avoid the risk of postpartum endometritis.

## Conclusion

The uterine inversion is a rare obstetric emergency. The strict respect of the steps of AMTSL would avoid this serious obstetrical complication. The use of intrauterine balloon tamponade is used to stop bleeding related to uterine atony and prevent the re-inversion.
